# Impact of Modified Blumgart Anastomosis on Pancreatic Fistula and Pancreaticojejunostomy Time During Laparoscopic Pancreaticoduodenectomy: Single-Center Experience

**DOI:** 10.3390/jcm14010090

**Published:** 2024-12-27

**Authors:** Jong Woo Lee, Jae Hyun Kwon, Jung-Woo Lee

**Affiliations:** Department of Surgery, Hallym University Sacred Heart Hospital, Anyang 14068, Republic of Korea; jongw.lee0212@gmail.com (J.W.L.); ponakwon@gmail.com (J.H.K.)

**Keywords:** pancreaticojejunostomy, laparoscopic, pancreaticoduodenectomy, anastomosis, POPF

## Abstract

**Background**/**Objectives**: The aim of this study is to evaluate the impact of modified Blumgart anastomosis methods during pancreaticojejunostomy (PJ) on the incidence of clinically relevant postoperative pancreatic fistula (POPF) after laparoscopic pancreaticoduodenectomy (LPD). **Methods**: This is a retrospective cohort study analyzing data of patients who underwent LPD from 2018 to 2022. The primary endpoint was the incidence of grade B and C POPF based on the International Study Group on Pancreatic Fistula criteria and PJ anastomosis time. Incidence of postoperative complications (Clavien–Dindo classification grade ≥ III) was also investigated. **Results**: A total of 148 patients, 99 patients in a modified Blumgart group and 49 patients in a continuous suture group, were enrolled. There were no statistically significant differences in the general and intraoperative characteristics found between the two groups (*p* > 0.05). There was no significant difference in pancreas texture (*p* = 0.397) and diameter of pancreatic duct (*p* = 0.845). Grade B and C POPF occurred in five patients (5.1%) in the modified Blumgart group and three patients (6.1%) in the continuous suture group with no statistical difference (*p* = 0.781). A total of eleven patients (11.1%) in the modified Blumgart group and four patients (8.2%) in the continuous suture group had postoperative complication (Clavien–Dindo Classification grade 3 or more). Mortality within 90 days was 2 (2%) and 0 (0%), respectively. The PJ anastomosis times in the modified Blumgart group and continuous suture group were 28.8 ± 5.94 min and 35 ± 7.71 min, respectively (*p* = 0.003). **Conclusions**: This study suggests that modified Blumgart PJ showed shorter anastomosis time with comparable outcome to continuous suture methods in LPD.

## 1. Introduction

Pancreaticoduodenectomy (PD) is a cornerstone surgical procedure for benign or malignant diseases of the periampullary region [1,2]. Despite its widespread application, PD remains one of the most technically demanding operations because of postoperative complications and mortality. PD is associated with various complications—postoperative pancreatic fistula (POPF), post-pancreatectomy hemorrhage (PPH), delayed gastric emptying (DGE), bile, or chyle leak. The incidence of postoperative complications was reported as 30–50%, especially that of POPF which was reported to be 3–45% and with a related mortality rate of 3–8% [3,4]. POPF is important because it can lead to intra-abdominal abscess, sepsis, hemorrhage, and life-threatening conditions with mortality up to 40% [5].

There have been various attempts to reduce the occurrence of POPF. They include studies related to the use of octreotide or fibrin sealants to pancreatic remnant, internal or external pancreatic duct stenting, pancreaticogastrostomy (PG), pancreaticojejunostomy (PJ) anastomosis, duct-to-mucosa (DTM), and invagination dunking methods. Despite these efforts, the best anastomosis procedure for pancreatic duct reconstruction is controversial; DTM anastomosis with an internal stent at the PJ anastomosis is the preferred choice for most surgeons [6,7,8,9].

Pancreatic DTM anastomosis facilitates rapid and effective healing by preventing exposure to pancreatic fluid, enabling the anastomotic stoma to heal quickly. Additionally, during PJ anastomosis, the remnant pancreatic parenchyma is enclosed by the seromuscular layer, which minimizes the risk of bleeding and helps maintain the long-term patency of the pancreatic duct. As a result, DTM is widely recommended by pancreatic surgery experts globally [6,7,8,9]. However, DTM PJ anastomosis has certain limitations: a potential dead space may remain between the pancreatic resection surface and the jejunal wall, leading to fluid retention. Moreover, performing pancreatic DTM anastomosis is particularly challenging in cases with a small diameter of pancreatic duct or thin pancreatic parenchyme.

Recently, DTM PJ anastomosis has become the preferred choice in many centers. For the seromuscular suture technique, the interrupted or continuous outer-layer suture of the pancreatic capsule, referred to as the “Cattell-Warren” technique, has traditionally been widely used. Recently, the Blumgart technique or its modifications are increasingly adopted following reports highlighting its advantages [10,11,12,13,14,15,16,17].

The interrupted or continuous anastomosis involves suturing the anterior and posterior layers through the pancreatic capsule and seromuscular layer of the jejunum. In contrast, the Blumgart method utilizes transpancreatic sutures tensioned with the seromuscular layer of the jejunum. By minimizing the number of sutures, this method reduces damage to the pancreatic parenchyma. The Blumgart method aims to create an invagination within the small bowel, encapsulating the remnant pancreas, with a DTM anastomosis positioned on the antimesenteric side of the bowel. This approach prevents pancreatic tearing and alleviates tension on the DTM anastomosis.

Several studies have demonstrated the effectiveness and feasibility of the Blumgart technique, although most have not reached definitive conclusions. Meanwhile, minimally invasive pancreaticoduodenectomy (MIPD)—including laparoscopic pancreaticoduodenectomy (LPD) and robotic pancreaticoduodenectomy (RPD)—has been increasingly adopted in recent years. MIPD offers potential benefits such as reduced postoperative pain, shorter hospital stays, and faster recovery times. However, it presents significant technical challenges, particularly in ensuring safe and efficient anastomosis techniques.

Given this trend, further research is necessary in the field of minimally invasive surgery. This study aims to evaluate the perioperative outcomes of the modified Blumgart technique compared to the conventional continuous suture in patients undergoing LPD.

### 1.1. Surgical Procedures

#### 1.1.1. Modified Blumgart Methods

In the first step of this technique, interrupted 3-0 polypropylene sutures (approximately 20 cm in length) are placed anteroposteriorly through the full thickness of the pancreas and the seromuscular layer of the jejunum, parallel to the long axis of the jejunum. The suture is then advanced back through the full thickness of the pancreas and tied down, leaving the needle on. On the cranial to the pancreatic duct, typically one or two transpancreatic sutures are performed.

Next, the DTM anastomosis is performed before placing transpancreatic sutures caudal to the pancreatic duct due to limited exposure and dexterity in the laparoscopic field. An enterotomy is created in the jejunum directly opposite the pancreatic duct. The DTM anastomosis incorporates the pancreatic duct, pancreatic parenchyma, and the full thickness of the jejunum. Typically, four to six interrupted 5-0 PDS sutures are used, beginning at the 9 o’clock, 3 o’clock, and 6 o’clock positions. Each posterior suture is tied and cut as the anastomosis progresses.

According to the size of the pancreatic duct, a silastic tube (ranging from 2 mm to 3.5 mm) is used as an internal stent, inserted into the pancreatic duct and jejunum. The stent is fixed with a 6 o’clock position suture, after which the anterior side of the duct-to-mucosa anastomosis is completed. Once the DTM anastomosis is finished, one or two additional transpancreatic sutures are placed caudal to the main pancreatic duct and tied. Using the needles from the posterior wall, the anterior side of the jejunum is sutured, taking care to avoid injury to the pancreatic duct. The pancreatic stump is fully enclosed by the jejunum.

**Figure 1:**
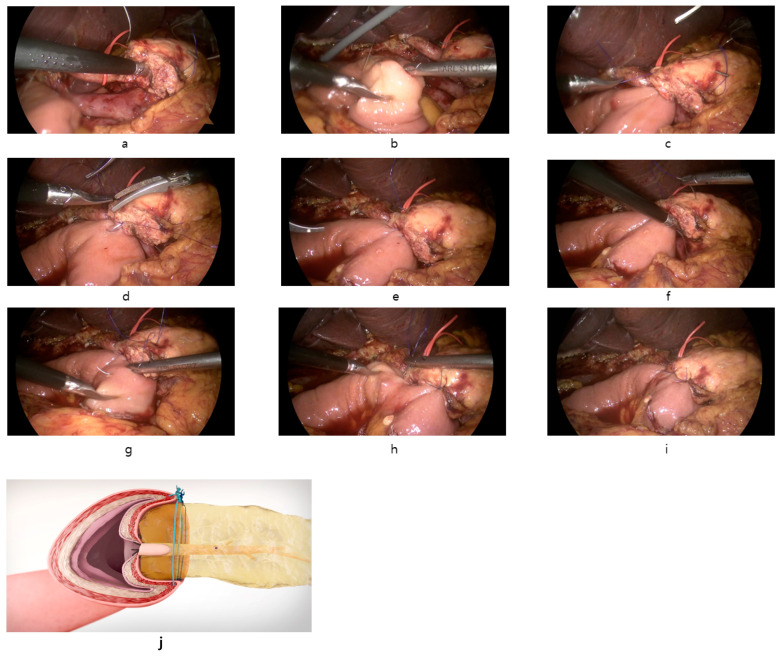
Intraoperative images of the reconstruction of PJ anastomosis with modified Blumgart technique in LPD. (**a**): Transpancreatic suture through full-thickness pancreas to seromuscular jejunum using 3-0 Prolene with needle straightened. (**b**): Suture of posterior seromuscular wall of jejunum parallel to the long axis of the jejunum. (**c**): Tie the suture and approximation of jejunum to pancreas. (**d**): Opening of jejunum and duct-to-mucosa anastomosis with internal stent. 5-0 PDS interrupted suture was used. (**e**): Completion of duct-to-mucosa anastomosis. (**f**): Additional full-thickness pancreas suture caudal to pancreatic duct (**g**): Suture of anterior seromuscular wall of jejunum perpendicular to the long axis of the jejunum. (**h**): Anterior seromuscular jejunum suture cranial to pancreatic duct. (**i**): Completion of modified Blumgart pancreaticojejunostomy after interrupted reinforcing suture of anterior side. (**j**): Coronal view after modified Blumgart PJ anastomosis.

Finally, interrupted reinforcement sutures are placed on the anterior side of the pancreatic duct to ensure a secure anastomosis (Figure 1).

#### 1.1.2. Conventional Continuous Suture (2-Layer)

The outer layer of the PJ on the dorsal side is started with continuous suturing of the pancreatic capsule at the cranial edge of the resection surface to the seromuscular layer of the jejunum using 4-0 polypropylene. The suture is continued to the caudal direction until reaching the caudal edge. At this stage, the tension on the suture is maintained to ensure the pancreas and jejunum are tightly adhered.

Next, an opening is created in the jejunum, and a DTM anastomosis is performed following the method described in the modified Blumgart technique. The ventral layer of the pancreatic capsule and the anterior side of the jejunum are sutured using 4-0 polypropylene in a continuous manner.

## 2. Materials and Methods

### 2.1. Study Design and Participants

This is a retrospective cohort study. Patients diagnosed with benign or malignant disease of pancreas, bile duct, ampulla of Vater, and duodenum were analyzed. This study includes 148 patients who underwent LPD from January 2018 to December 2022 at the Department of General Surgery of the Hallym University Sacred Heart Hospital, Anyang, Republic of Korea.

This retrospective study was conducted in accordance with the Helsinki Declaration and International Ethical Guidelines for Biomedical Research Involving Humans. The study was approved by the Medical Ethics Committee of Hallym University Sacred Heart Hospital (approval number: HALLYM 2023-03-004-001).

### 2.2. Data Collection and Definitions of Postoperative Complications

The general clinicopathological factors (age, sex, body mass index, ASA classification, pathological diagnosis), intraoperative factors (operative procedure, superior mesenteric vein or portal vein resection, pancreas texture operative time) and postoperative results (POPF, postoperative complications, hospital stay, mortality) of all patients were collected and analyzed retrospectively. Complications after laparoscopic PD include POPF, postoperative bleeding, bile leak, chyle leak, abdominal fluid collection, lung complication, portal vein thrombus, anastomosis stricture, small bowel ileus, and afferent loop syndrome. POPF was defined and graded according to the 2016 update of the International Study Group of Pancreatic Surgery (ISGPS) [3]. Grade B and grade C POPF were considered clinically relevant (CR-POPF). DGE and PPH were also defined according to the ISGPS criteria. The classification of postoperative complications followed the Clavien—Dindo classification 2004 [18]. Complications graded ≥ III were considered as severe complications. Postoperative mortality was defined as a death within 90 days after operation.

### 2.3. Operative Techniques for Pancreaticojejunostomy

The detailed procedure of modified Blumgart and continuous suture was described in the Introduction Section. In our department, several modifications were made from the original Blumgart technique to make it suitable for laparoscopic surgery. For seromuscular suture, straightened 3-0 polypropylene single-armed needles were used. Knots for approximation of posterior and anterior jejunum were made on the anterior surface of the pancreas. Another point is that DTM anastomosis is performed before transpancreatic sutures caudal to the pancreatic duct to secure better visibility and space.

### 2.4. Postoperative Management

One or two drainage tubes were routinely placed above and below the PJ anastomosis in each patient. The color and amount of the drainage fluid were recorded every postoperative day and drainage fluid amylase and lipase were checked every day until the fifth postoperative day. Postoperative CT was performed on the fifth postoperative day. The drainage tube was removed if there was no abnormal fluid collection around the PJ anastomosis and the drainage amylase level was normal. If, in the patient with drainage, amylase levels did not increase, sips of water were initiated starting from the second postoperative day, considering the patient’s recovery status and tolerability. Subsequently, the oral diet progression was gradually advanced to a liquid diet and then a soft diet considering the recovery of gastrointestinal function.

### 2.5. Statistical Analysis

All statistical analyses were performed using IBM SPSS statistic version 22.0 (IBM Corp., Armonk, NY, USA). In patient demographics, perioperative, and postoperative factors, categorical variables were analyzed using the chi-square test of independence or the Fisher’s exact test to determine whether there was an association between the variables in the two groups. Continuous variables were compared using Student’s *t*-test and expressed as mean ± standard deviation. Kolmogorov—Smirnov test and Shapiro–Wilk test were used to check the normal distribution. It was considered statistically significant when *p* < 0.05. Statistically significant variables demonstrated in univariate analysis were incorporated in multivariate logistic regression to analyze the independent risk factors for pancreatic fistula after PJ.

## 3. Results

### 3.1. Patient Demographics and Baseline Clinical Data

A total of 148 patients were included in this study, with 99 patients in the modified Blumgart group and 49 patients in the conventional continuous suture group. The baseline characteristics of the enrolled patients are summarized in Table 1. There were no statistically significant differences in terms of age, sex, body mass index, ASA classification, previous operation history, diabetes, hypertension, hepatitis neoadjuvant chemotherapy, disease location, and malignancy (*p* > 0.05). However, the proportion of preoperative biliary drainage was significantly higher in the modified Blumgart group (46.5% vs. 28.6%, *p* = 0.037).

### 3.2. Operative Outcomes

The intraoperative factors are listed in Table 2. In the operative data, the proportion of pylorus preserving pancreaticoduodenectomy was similar in the two groups (92.9% vs. 91.8%). Portal vein or superior mesenteric vein resection was performed in the conventional continuous suture group with significance (22.4% vs. 10.1%, *p* = 0.043). The soft pancreas (22.2% vs. 28.6%) and diameter of the pancreatic duct (2.22 ± 0.66 mm vs. 2.25 ± 0.57 mm) showed no difference (*p* > 0.05). Total operative time (319.11 ± 94.59 min vs. 411.1 ± 96.68 min, *p* < 0.001) and PJ anastomosis time (28.8 ± 5.94 vs. 35 ± 7.71 min, *p* = 0.003) were significantly shorter in the modified Blumgart method group. Estimated blood loss showed no significant difference (*p* > 0.05).

### 3.3. Postoperative Outcomes and Complications

**Figure 2:**
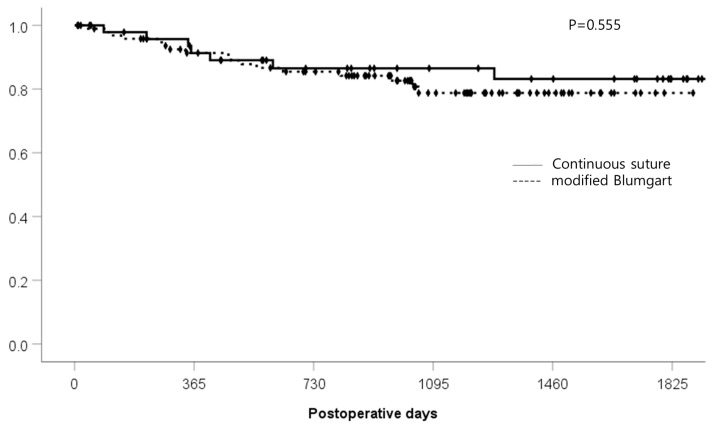
Postoperative follow-up period (days).

The median follow-up periods for the continuous suture group and the modified Blumgart group were 1395 (46–2397) days and 1009 (9–1889) days, respectively. Kaplan—Meier analysis of the follow-up periods between the two groups showed no significant difference (*p* = 0.555) (Figure 2).

When comparing the postoperative complications, the incidence CR-POPF was comparable between the groups (5.1% vs. 6.1%, *p* = 0.786). Notably, portal vein thrombus was more frequent in the continuous suture group (6.1% vs. 0%, *p* = 0.035). The mean postoperative hospital stay was slightly longer in the continuous suture group (15.55 ± 7.89 days) compared to the modified Blumgart group (13.73 ± 7.3 days), though this difference did not reach statistical significance (*p* = 0.166). Mortality rates were low overall (2% in modified Blumgart vs. 0% in continuous suture) and severe complications did not show a significant difference between the two groups (*p* = 0.576). There were two reoperation cases in the modified Blumgart group: one underwent a gastrojejunal anastomosis revision, and the other underwent reoperation for afferent loop obstruction due to internal herniation. The other reoperation case in the continuous suture group was PJ revision due to POPF. No significant differences were observed in other complications, such as postoperative bleeding, bile leak, chyle leak, fluid collection, lung complication, CJ, GJ stricture, or small bowel ileus (Table 3).

### 3.4. Risk Factors of Postoperative Pancreatic Fistula

Logistic regression analysis was performed to investigate the factors associated with postoperative pancreatic fistula (Table 4). In the univariate analysis, soft pancreas texture (odds ratio [OR], 4.355; 95% confidence interval [CI], 1.102–17.208; *p* = 0.036) was a significant factor for CR-POPF. In the multivariate analysis, PJ time (OR, 1.532; 95% CI, 1.011–2.32; *p* = 0.044) was the only independent risk factor for CR-POPF. The PJ technique was not related to POPF. Additionally, a logistic regression analysis was conducted to identify risk factors for postoperative complications of Clavien—Dindo classification grade 3 or higher, but no independent risk factors were identified.

## 4. Discussion

In this retrospective study comparing the modified Blumgart anastomosis with the conventional continuous suture in laparoscopic pancreaticoduodenectomy, we observed that the modified Blumgart technique resulted in significantly shorter total operative time (319.11 ± 94.59 min vs. 411.1 ± 96.68 min, *p* < 0.001) and PJ time (28.8 ± 5.94 vs. 35 ± 7.71 min, *p* = 0.003) compared to the conventional continuous suture method. Additionally, the incidence of CR-POPF, overall complications, and mortality were comparable between the two groups (*p* > 0.05). Regarding the risk factors of CR-POPF, the logistic regression analysis showed that PJ time was the only independent risk factor (*p* = 0.044). In addition, the PJ anastomosis type, pancreas texture, and pancreatic duct diameter were not shown to be significant factors for POPF (*p* > 0.05).

The minimal approach to hepatobiliary and pancreatic surgery has become a trend. Several studies have reported that LPD and RPD have the advantages of less bleeding, less postoperative pain, and short postoperative recovery time compared to open pancreaticoduodenectomy [19,20]. Due to these advantages, despite the complex surgical procedure and life-threatening risk, more centers are adopting minimally invasive pancreaticoduodenectomy (MIPD). In fact, POPF can lead to life-threatening conditions such as PPH and infectious complications, making it a significant concern for surgeons. Sometimes, this concern is highly stressful and serves as a hurdle for initiating MIPD.

The risk factors for POPF are known to include age, BMI, tumor type, pancreatic texture, main pancreatic duct diameter, intraoperative blood loss, and PJ anastomosis technique [21,22]. Among them, PJ anastomosis is the only factor that the surgeon can control. Reviewing previous studies on POPF after mostly OPD in recent years, the best anastomosis of pancreas stump and digestive tract remains controversial. However, many reports have suggested that DTM anastomosis with internal stent at PJ anastomosis may ensure complete pancreatic juice drainage and maintain the long-term patency of the pancreatic anastomosis. Regarding the seromuscular anastomosis technique combined with DTM, there have been several studies reporting lower incidence of POPF in the Blumgart technique ranging from 2.5% to 20.5%, demonstrating its safety, feasibility, and superiority compared to the interrupted suture technique [13]. Based on these study results, the Blumgart technique has been widely used in PD to date. Recently, several studies reporting on the usefulness of Blumgart anastomosis in LPD and RPD have been published [23,24].

Shear forces at the pancreas have been considered as an important cause of POPF. The Blumgart technique could eliminate tangential tension and reduce force at the pancreatic stump, which helps to prevent the fragile pancreas from being cut through [25]. Compared to the original Blumgart method, the unique aspect of our modification is the use of a single-arm needle to tie knots on the anterior surface of the pancreas, ensuring the close approximation of both the posterior and anterior walls of the jejunum. We also reduced the number of transpancreatic sutures from two to four depending on the cranio-caudal length of pancreas. In addition, to secure a better view, enterotomy and DTM anastomosis were performed first before the caudal part of the transpancreatic suture. These aspects were modified to suit the laparoscopic view and setting. This technique is simple and could make it possible to reduce anastomosis time significantly. The criteria for an ideal PJ technique are a low incidence of POPF and complication, a wide application range, and being easy to learn. According to previous studies, important points of the PJ technique to prevent POPF include complete drainage of pancreatic juice, good blood flow in the pancreatic stump, prevention of pancreatic parenchyma laceration, and close contact of jejunum wall with pancreatic cut surface [13]. In our study results, CR-POPF rate (5.1%), total operation time (319.11 ± 94.59 min), and PJ anastomosis time (28.8 ± 5.94 min) appear to be acceptable compared to previous studies performed in LPD [26,27]. The postoperative follow-up periods were sufficient (1395 (46–2397), 1009 (9–1889) days, respectively), and there were no reoperation cases due to PJ anastomosis issues in the modified Blumgart group. These results also support long-term safety. Considering these points, the modified Blumgart technique appears to satisfy the ideal criteria mentioned above.

From the perspective of time consumption, the modified Blumgart technique is considered particularly useful in LPD with longer operation times. The findings suggest that the modified Blumgart technique may offer certain time-saving advantages without compromising patient safety. Our hypothesis was that the modified Blumgart method could reduce POPF incidence by reducing the laceration of the pancreatic parenchyma and more completely covering the pancreatic cut surface. Although it did not statistically significantly reduce the incidence of POPF compared to the conventional continuous suture method, this study may contribute to the current literature by providing evidence regarding the optimal pancreatic anastomosis technique in a laparoscopic setting. By focusing on a laparoscopic context, this research provides essential insights into optimizing surgical techniques for MIPD, addressing a critical gap in current surgical practice.

The strengths of our study include a relatively large cohort size and a standardized laparoscopic surgical approach. Nevertheless, our study has several imitations. First, this study has a single-center retrospective nature with possible cofounding factors. Thus, it may lack predictability and randomness, potentially leading to biases. However, all patients underwent surgery by a single skilled surgeon with a consistent surgical technique reducing possible variations, which might minimize bias. Second, conversely, all procedures were performed by a single skilled surgeon, which may limit the generalizability of the results. Before starting this study, he had experience with over 200 OPD and more than 50 LPD cases.

## 5. Conclusions

We described several modifications to the original Blumgart anastomosis for laparoscopic setting. Our technique was associated with acceptably low rates of CR-POPF and showed noninferior outcomes and long-term patency compared to continuous suture in LPD.

In conclusion, the modified Blumgart anastomosis technique in LPD appears to be safe, feasible, and effective with reduced operative and PJ times compared to the traditional continuous suture technique. Further prospective, randomized studies are needed to confirm these findings and to establish standardized guidelines for anastomosis techniques in laparoscopic pancreatic surgery.

## Figures and Tables

**Table 1:** Preoperative and pathological results.

	Modified Blumgart (*n* = 99)	Continuous Suture (*n* = 49)	*p*-Value
Age (yr)	68.18 ± 10.77	67.18 ± 12.87	0.255
Male sex	49 (49.5%)	20 (40.8%)	0.319
Body mass index (kg/m^2^)	23.35 ± 3.28	24.53 ± 3.3	0.549
ASA PS classification			0.167
ASA 1	6 (6.1%)	0 (0)	
ASA 2	42 (42.4%)	17 (34.7%)	
ASA 3	48 (48.5%)	31 (63.3%)	
ASA 4	3 (3%)	1 (2%)	
Previous upper abdominal surgery	12 (12.1%)	2 (4.1%)	0.144
Medical history			
Diabetes mellitus	19 (19.2%)	7 (14.3%)	0.460
Hypertension	28 (28.3%)	13 (26.5%)	0.823
Hepatitis	2 (2%)	0 (0)	1.000
Neoadjuvant Chemo	0(0)	1 (2)	0.331
Preoperative biliary drainage	46 (46.5%)	14 (28.6%)	0.037
Location of disease			0.444
Pancreas head	46 (46.5%)	28 (57.1%)	
Ampulla of Vater	20 (20.2%)	9 (18.4%)	
Duodenum	6 (6.1%)	4 (8.2%)	
Distal CBD	27 (27.3%)	8 (16.3%)	
Malignancy			0.791
Malignant	79 (79.8%)	40 (81.6%)	
Benign	20 (20.2%)	9 (18.4%)	

ASA: American society of Anesthesiologists, PS: performance status, Chemo: chemotherapy, CBD: common bile duct.

**Table 2:** Intraoperative data.

	Modified Blumgart (n = 99)	Continuous Suture (n = 49)	*p*-Value
Operative procedure			1.000
Whipple procedure	7 (7.1%)	4 (8.2%)	
PPPD	92 (92.9%)	45 (91.8%)	
SMV and/or PV resection	10 (10.1%)	11 (22.4%)	0.043
Pancreas texture, soft	22 (22.2%)	14 (28.6%)	0.397
Diameter of main pancreatic duct (mm)	2.22 ± 0.66	2.25 ± 0.57	0.845
Total operative time (min)	319.11 ± 94.59	411.1 ± 96.68	<0.001
Time for PJ (min)	28.8 ± 5.94	35 ± 7.71	0.003
Estimated blood loss (mL)	430 ± 354.2	437.9 ± 368.1	0.310

PPPD: pylorus preserving pancreaticoduodenectomy, SMV: superior mesenteric vein, PV: portal vein, PJ: pancreaticojejunostomy.

**Table 3:** Postoperative outcomes including postoperative pancreatic fistula.

	Modified Blumgart (*n* = 99)	Continuous Suture (*n* = 49)	*p*-Value
Median follow up period, days	1395 (46–2397)	1009 (9–1889)	0.555
POPF			0.953
None	24 (24.2%)	10 (20.4%)	
Biochemical leak	70 (70.7%)	36 (73.5%)	
POPF B	4 (4%)	2 (4.1%)	
POPF C	1 (1%)	1 (2%)	
CR-POPF	5 (5.1%)	3 (6.1%)	0.786
PPH	1 (1%)	0	1.0
Bile leak	2 (2%)	0	1.0
Chyle leak	1 (1%)	0	1.0
Intraabdominal fluid collection or abscess	8 (8.1%)	4 (8.2%)	1.0
Lung complication	2 (2%)	0	1.0
PV thrombus	0	3 (6.1%)	0.035
CJ stricture	1 (1%)	0	1.000
GJ stricture	1 (1%)	0	1.000
Small bowel ileus	3 (3%)	0	0.551
A-loop syndrome	1 (1%)	0	1.000
Cerebral stroke	1 (1%)	0	1.000
Severe Complications (Clavien—Dindo ≥ III)	11 (11.1%)	4 (8.2%)	0.576
Postoperative hospital stay (days)	13.73 ± 7.3	15.55 ± 7.89	0.166
Reoperation	2 (2%)	1 (2%)	1.0
Mortality	2 (2%)	0	1.0

POPF: postoperative pancreatic fistula, CR-POPF: clinically relevant postoperative pancreatic fistula, PPH: postpancreatectomy hemorrhage, PV: portal vein, CJ: choledochojejunal anastomosis, GJ: gastrojejunal anastomosis.

**Table 4:** Univariate and multivariate analysis for clinically relevant postoperative pancreatic fistula.

Variable	Univariate Analysis		Multivariate Analysis	
	OR (95% CI)	*p*-Value	OR (95% CI)	*p*-Value
Age (yr)	1.013 (0.952–1.078)	0.688	0.772 (0.571–1.044)	0.093
Sex, male	2.413 (0.580–10.037)	0.226	NA	NA
Body mass index (kg/m^2^)	1.162 (0.967–1.396)	0.108	NA	NA
ASA PS classification (III, IV vs. I, II)	1.023 (0.263–3.973)	0.974	NA	NA
Diabetes mellitus	0.570 (0.068–4.765)	0.604	0	0.998
Hypertension	3.576 (0.910–14.053)	0.068	173,716.974 (0.587–4.395 × 10^10^)	0.057
Previous upper abdominal surgery	3.024 (0.564–16.212)	0.197	274.928 (0.667–113,352.415)	0.068
Preoperative biliary drainage	0.719 (0.173–2.995)	0.651	68.581 (0.480–9794.303)	0.095
Pancreas texture, soft	4.355 (1.102–17.208)	0.036	NA	NA
Diameter of main pancreatic duct (mm)	1.646 (0.658–4.118)	0.286	NA	NA
Total operative time (min)	1.003 (0.997–1.009)	0.262	NA	NA
Time for PJ (min)	1.058 (0.933–1.199)	0.378	1.532 (1.011–2.320)	0.044
Type of anastomosis, modified Blumgart	0.989 (0.237–4.135)	0.988	NA	NA

ASA PS: American society of Anesthesiologists performance status, PJ: pancreaticojejunostomy anastomosis, NA: not available.

## Data Availability

No new data were created or analyzed in this study. Data sharing is not applicable to this article.
